# Long-term protective efficacy of the *Escherichia coli-*produced HPV-16/18 bivalent human papillomavirus vaccine in women vaccinated at 18–45 years: A 9-year follow-up study

**DOI:** 10.1016/j.imj.2025.100164

**Published:** 2025-01-10

**Authors:** Xinhua Jia, Shangying Hu, Xuefeng Kuang, Youlin Qiao

**Affiliations:** a School of Population Medicine and Public Health, Chinese Academy of Medical Sciences and Peking Union Medical College, Beijing 100730, China; b Department of Epidemiology, National Cancer Center/National Clinical Research Center for Cancer/Cancer Hospital, Chinese Academy of Medical Sciences and Peking Union Medical College, Beijing 100021, China

**Keywords:** Human papilloma virus, Human papilloma virus infection, Human papilloma virus vaccine, Long-term follow-up, Protective efficacy

## Abstract

**Background:**

To assess the enduring protective efficacy of the recombinant human papilloma virus (HPV) 16/18 bivalent vaccine (produced in *Escherichia coli*) in preventing HPV infection.

**Methods:**

A long-term follow-up study was conducted in Xinmi, Henan Province, in September 2022, 9 years post-administration of the initial vaccine dose. This study was grounded in the phase III clinical trial of the vaccine (NCT01735006). Participants were recalled to collect exfoliated cervical cells for HPV DNA genotyping. The long-term protective efficacy of the vaccine against HPV infection was evaluated using Poisson distribution.

**Results:**

A total of 1 123 volunteers were recalled, comprising 558 individuals in the experimental group and 565 in the control group, with mean ages of 30.80 ± 7.33 years and 30.64 ± 7.51 years, respectively. At baseline (0 days before vaccination), 147 participants (13.09%) were infected with any type of HPV. By the ninth year of follow-up, the overall HPV infection rate within the entire cohort had increased to 16.65%. In the intention-to-treat analysis, the demonstrated protective efficacy against HPV-16, HPV-18, and HPV-16/18 was 83.12% (95% confidence interval [CI]: 24.20–98.17), 100.00% (95% CI: −10.50 to 100.00) and 87.34% (95% CI: 46.17–98.59), respectively. In the modified intention-to-treat analysis, the protective efficacy of the vaccine against HPV-16, HPV-18, and HPV-16/18 was 82.90% (95% CI: 23.20–98.14), 100.00% (−10.71 to 100.00), and 87.36% (95% CI: 46.20–98.59), respectively.

**Conclusions:**

Vaccination with the bivalent HPV vaccine offers long-term protection against HPV-16/18 infections for at least 9 years.

## Highlights

• Long-term efficacy assessment: evaluated the protective efficacy of the recombinant HPV 16/18 bivalent vaccine over a 9-year period post-vaccination.
• Phase III clinical trial follow-up: conducted a long-term follow-up study based on the phase III clinical trial (NCT01735006) in Xinmi, Henan Province, China.
• Significant protective efficacy: demonstrated high protective efficacy against HPV-16 and HPV-18 infections, exceeding 82% in both ITT and mITT analyses.
• Sustained protection against HPV-18: observed 100% protective efficacy against HPV-18 infection after nine years.
• Support for vaccine longevity: findings indicate the bivalent HPV vaccine provides sustained long-term protection against HPV-16/18 infections for at least nine years.

## 1 Introduction

Cervical cancer presents a significant global challenge to female health. In 2022, approximately 660 000 new cases of cervical cancer were diagnosed worldwide, resulting in around 350 000 deaths.^1^ In China, both the incidence and mortality rates of cervical cancer have increased in the past two decades,^2^ with a shift towards younger women. In May 2018, the World Health Organization (WHO) called for the elimination of cervical cancer through the use of human papilloma virus (HPV) vaccines combined with effective screening, treatment, and management strategies.^3^ The HPV vaccine has demonstrated high efficacy in preventing HPV infection and precancerous lesions.^4^ China approved the use of HPV vaccines only in 2016, approximately a decade later than countries where HPV vaccines were initially marketed.^4^

Currently, three types and five brands of HPV vaccines are available in China: bivalent, quadrivalent, and nine-valent. The bivalent vaccine offers protection against high-risk HPV-16 and HPV-18 infections, whereas the quadrivalent vaccine additionally provides coverage against low-risk HPV-6 and HPV-11. By contrast, the nonavalent vaccine extends protection to HPV types HPV-16, 18, 6, 11, 31, 33, 45, 52, and 58.^5^, ^6^, ^7^ A domestically developed bivalent vaccine targeting HPV-16/18, produced using *Escherichia coli*, has demonstrated high efficacy in preventing HPV-16/18 associated high-grade genital lesions and persistent infections in Chinese females aged 18–45 years, with sustained effectiveness observed up to 66 months post-initial vaccination.^8^ It was approved for marketing in China in 2019 and received World Health Organization Prequalification (WHO PQ) certification in 2021. However, evidence of its long-term protective efficacy is lacking, which is vital for HPV vaccination strategy and public confidence. To address this, we conducted a long-term follow-up study to evaluate the vaccine's protection against HPV infection 9 years post-vaccination.

## 2 Materials and methods

### 2.1 Participants and study design

The phase III study (NCT01735006) was a multicenter, double-blind, randomized, controlled trial assessing the efficacy of three-dose vaccination with *E. coli*-produced HPV-16/18 bivalent vaccine in Chinese women aged 18–45 years at first dose administration. It evaluated vaccine efficacy (VE) up to 72 months with a median follow-up of 5.5 years, concluding in 2019. Women were excluded if they were virgins; had more than four sexual partners during their lifetime; were pregnant or lactating; had previously been vaccinated against HPV 16 or HPV 18; had a history of any sexually transmitted disease or abnormal cervical cancer screening results within the past 2 years; suffered from immunological diseases that could affect the immune response, severe internal diseases, or other chronic diseases needing to be treated; or had a history of allergies to vaccinations. The study vaccine was jointly developed by Xiamen Innovax Biotech Co., Ltd. (INNOVAX), Xiamen University, and Beijing Wantai Biological Pharmacy Enterprise Co., Ltd. The vaccine was inspected by the National Institutes for Food and Drug Control to conform to the Regulation on Manufacturing and Inspection of Recombinant Human Papillomavirus 16/18 Bivalent Vaccine (*E. coli*) (Draft) (https://www.duyaonet.com/_FileUpload/20240929/09291709459DD3DC1664E7074B.pdf) and was used within its validity period. Details of patient characteristics, study methods, and clinical trial results are published.^8^^,^^9^ The present long-term follow-up (LTFU) study was an open-labeled controlled study conducted at one of five sites of the initial study (Xinmi, Henan Province) in September 2022. All healthy and non-pregnant participants involved in the initial study were invited to the LTFU study. This study was approved by the Institutional Review Board of the Chinese Academy of Medical Sciences and Peking Union Medical College (No. CAMS & PUMC-IEC-2022-022) with written informed consent obtained before any surveys or examinations.

### 2.2 *Follow-up procedure*

Before the LTFU study, all participants were contacted by phone to introduce the study and obtain preliminary informed consent. During the LTFU visit, participants first underwent the informed consent process before proceeding with registration, questionnaire surveys, and gynecological examinations. After completing the routine gynecological examination, a cytobrush was inserted into the cervical os to collect samples from the transformation zone, cervical os, and along the cervical canal lesions, rotating it five times in one direction. The cytobrush was immediately placed into the preservation bottle and sent to the laboratory for HPV DNA testing.

### 2.3 *HPV DNA laboratory testing*

HPV DNA testing was conducted at the Central Laboratory of the Cancer Hospital of the Chinese Academy of Medical Sciences (Department of Cancer Epidemiology). A 1 mL aliquot of the sample was taken from the 20 mL Thinprep® PreservCyt®(Hologic Inc., Marlborough, MA 01752 USA) Solution for HPV DNA testing. DNA extraction was performed using the Total Nucleic Acid Isolation Kit and a MagNA Pure machine (Dynamax Biotech Co., Ltd., Gumei Rd., Shanghai). The baseline HPV DNA test was conducted using the SPF10-PCR-DEIA method. Specimens that tested positive for DEIA underwent HPV genotyping testing using reverse hybridization SPF10-LiPA25 (LiPA) and HPV-16/18 type-specific PCR-DEIA methods (TS-16/18). SPF10-LiPA25 tested 25 different HPV genotypes, including 14 oncogenic types (16, 18, 31, 33, 35, 39, 45, 51, 52, 56, 58, 59, 66, and 68) and 11 non-oncogenic types (6, 11, 34, 40, 42, 43, 44, 53, 54, 70, and 74).

During the LTFU stage, HPV DNA genotyping was performed using multiplex PCR and capillary electrophoresis (Health-25). This kit was designed with specific primers targeting the oncogenic genes E6/E7 in the HPV genome, enabling the specific amplification and capillary electrophoretic separation of different HPV genotypes. On the basis of the varying lengths of the specific amplified fragments, 25 HPV genotypes (including 14 oncogenic types [HPV 16, 18, 31, 33, 35, 39, 45, 51, 52, 56, 58, 59, 66, and 68] and 11 non-oncogenic types [HPV 6, 11, 26, 42, 43, 44, 53, 73, 81, 82, and 83) could be simultaneously typed in a single test. The SPF10-LiPA25 test and the health-25 test have shown good consistency in previous studies.^10^

### 2.4 *Statistical methods*

The formula used for calculating the long-term protective efficacy of the HPV-16/18 vaccine was

(1) $$\pi_{e}=1-\frac{e_{v}/\tau_{v}}{e_{p}/\tau_{p}}$$

where $e_{v}$ and $e_{p}$ are the number of HPV infection cases in the vaccine and control group, respectively, and $\tau_{v}$ and $\tau_{p}$ are the total number of people in the vaccine and control group, respectively. Assuming that the occurrence numbers of the two trial groups are independently Poisson-distributed, based on the total number of events

(2) $$e=e_{v}+e_{p}$$

the distribution of the HPV vaccine group's event number is binomial (e,p), where

(3) $$p=\frac{1-\pi }{2-\pi }$$

On the basis of the observed event occurrence numbers of the two trial groups (assuming $e_{v}<e_{p}$), the maximum $p_{1}$ that satisfies

$$p\left[n<e_{v}|\text{Bin}\left(e,p_{1}\right)\right]\leq 0.025$$

and the maximum $p_{h}$ that satisfies

$$p\left[n<e_{v}|\text{Bin}\left(e,p_{h}\right)\right]\leq 0.025$$

can be obtained. From $p_{1}$ and $p_{h}$, $\pi_{eh}$ and $\pi_{e1}$ were obtained. The 95% exact confidence interval for the protective efficacy of HPV infection was estimated as $\left[\pi_{e1},\;\;\pi_{eh}\right]$.

This study used baseline data and 2022 follow-up results from the cohort, excluding cases from the interim follow-up period. The dataset for analyzing the long-term protective efficacy of the vaccine was categorized into the intention-to-treat (ITT) set, the modified intention-to-treat (mITT) set, and the baseline HPV DNA-positive analysis set. The ITT set was defined as participants who received at least one dose of the vaccine and completed effective follow-up in 2022; the mITT group included participants who received at least one vaccine dose, completed follow-up in 2022, and were HPV DNA-negative at day 0. The baseline HPV DNA positive analysis set comprised individuals with any positive HPV type at baseline. Statistical analyses were conducted using R 4.2.1 and SAS 9.4, with a two-sided significance level of 0.05.

## 3 Results

**Fig. 1:**
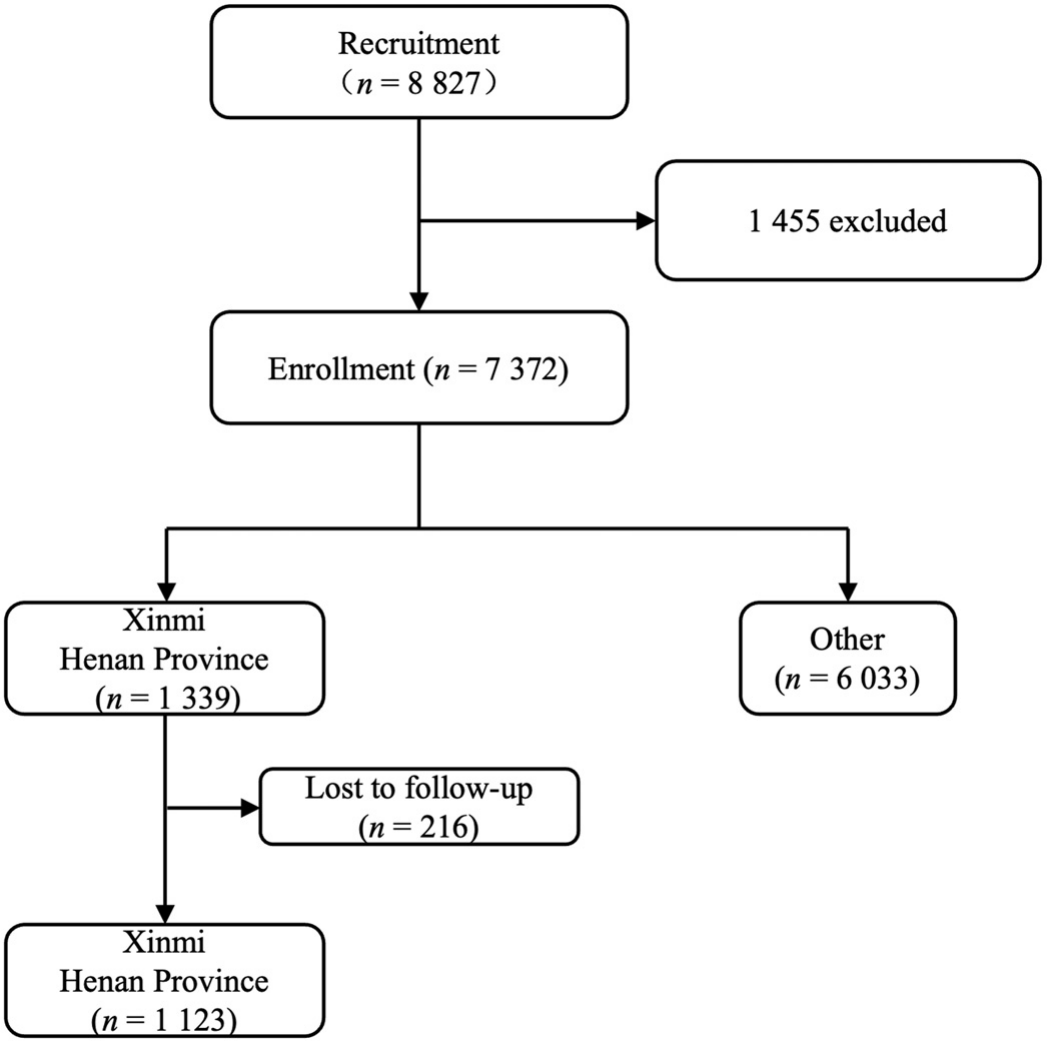
LTFU study flowchart. *Abbreviations*: LTFU, long-term follow-up.

The clinical trial enrolled a total of 1 339 participants from Xinmi, Henan Province, and completed enrollment in March to April 2013. During the long-term follow-up conducted in 2022, a total of 1 123 participants completed the follow-up process and were subsequently included in the analysis (Fig. 1). This cohort consisted of 558 individuals in the experimental group and 565 individuals in the control group, with mean ages of 30.80 ± 7.33 years and 30.64 ± 7.51 years, respectively. At baseline (0 days before vaccination), 147 participants (13.09%) were identified to have infections from any HPV type, with infection rates for HPV-16 and -18 recorded at 2.40% and 0.45%, respectively. In the ninth year of follow-up, the overall HPV infection rate of the entire cohort was 16.65%, with infection rates for HPV-16 and 18 recorded at 1.25% and 2.45%, respectively.

### 3.1 The protective efficacy in the ITT/mITT populations

In the ITT population, the bivalent HPV vaccine demonstrated a protective efficacy of 83.12% (95% confidence interval [CI]: 24.20–98.17) against HPV-16 infection, 100.00% (95% CI: −10.50 to 100.00) against HPV-18 infection, 87.34% (95% CI: 46.17–98.59) against HPV-16/18 infection, and 67.45% (95% CI: 29.06–86.49) against HPV-16, 18, 31, 33, 45 infection (Table 1). In the mITT population, which excluded individuals positive for the corresponding HPV types at baseline, the vaccine's protective efficacy was 82.90% (95% CI: 23.20–98.14) against HPV-16 infection, 100.00% (95% CI: −10.71 to 100.00) against HPV-18 infection, 87.37% (95% CI: 46.26–98.59) against HPV-16/18 infection, and 63.83% (95% CI: 23.30–84.33) against HPV-16, 18, 31, 33, 45 infection (Table 2).

**Table 1:** Efficacy of HPV-related infections (ITT).

HPV type	Control group (*n* = 565)		Vaccine group (*n* = 558)		Vaccine efficacy (95% CI^a^, %)
	Number of cases	Rate (%)	Number of cases	Rate (%)	
HPV-16	12	2.12	2	0.36	83.12 (24.20–98.17)
HPV-18	5	0.88	0	0.00	100.00 (−10.50–100.00)
HPV-16/18	16	2.83	2	0.36	87.34 (46.17–98.59)
HPV-31	8	1.42	3	0.54	62.03 (−58.20–93.51)
HPV-33	2	0.35	4	0.72	−102.51 (−2 138.71–70.98)
HPV-35	1	0.18	3	0.54	−203.76 (−15 846.78–75.61)
HPV-39	3	0.53	4	0.72	−35.01 (−821.64–77.16)
HPV-45	2	0.35	1	0.18	49.37 (−872.50–99.15)
HPV-51	6	1.06	11	1.97	−85.63 (−511.31–37.07)
HPV-52	12	2.12	23	4.12	−94.07 (−328.98–7.32)
HPV-56	9	1.59	6	1.08	32.50 (−112.35–80.23)
HPV-58	5	0.88	9	1.61	−82.26 (−592.29–45.15)
HPV-59	3	0.53	2	0.36	32.50 (−489.28–94.36)
5 HR-HPV types^b^	28	4.96	10	1.79	67.45 (29.06–86.49)
7 HR-HPV types^c^	44	7.79	39	6.99	10.25 (−41.35–43.21)
8 HR-HPV types^d^	45	7.96	41	7.35	7.75 (−44.08–41.08)
13 HR-HPV types^e^	72	12.74	67	12.01	5.78 (−33.28–33.46)
Any type	98	17.35	89	15.95	8.04 (−23.79–31.77)

a Confidence intervals (CI) were calculated using the two-step approach, which did not account for dependence.

b 5 HR-HPV types: 16, 18, 31, 33, 45.

c 7 HR-HPV types: 16, 18, 31, 33, 45, 52, 58.

d 8 HR-HPV types: 16, 18, 31, 33, 35, 45, 52, 58.

e 13 HR-HPV types: 16, 18, 31, 33, 35, 39, 45, 51, 52, 56, 58, 59, 68. *Abbreviations*: ITT, intention-to-treat; HPV, human papilloma virus.

**Table 2:** Efficacy of HPV-related infection protection (mITT).

HPV type	Control group			Vaccine group			Vaccine efficacy (95% CI^a^, %)
	Total number	Number of cases	Rate (%)	Total number	Number of cases	Rate (%)	
HPV-16	555	12	2.16	541	2	0.37	82.90 (23.20–98.14)
HPV-18	563	5	0.89	555	0	0.00	100.00 (−10.71–100.00)
HPV-16/18	564	16	2.84	558	2	0.36	87.37 (46.26–98.59)
HPV-31	565	8	1.42	555	3	0.54	61.82 (−59.06–93.48)
HPV-33	562	2	0.36	553	4	0.72	−100.62 (−2 146.95–70.87)
HPV-35	562	1	0.18	555	3	0.54	−103.78 (−15 852.11–75.60)
HPV-39	564	3	0.53	553	6	1.08	−103.98 (−1 160.51–56.44)
HPV-45	565	2	0.35	557	1	0.18	49.28 (−874.24–99.14)
HPV-51	555	10	1.80	550	10	1.82	−0.91 (−170.13–62.31)
HPV-52	550	12	2.18	539	22	4.08	−87.07 (−314.73–11.35)
HPV-56	561	9	1.60	553	5	0.90	43.63 (−87.31–85.16)
HPV-58	560	5	0.89	557	9	1.62	−80.97 (−587.39–45.52)
HPV-59	565	5	0.88	556	2	0.36	59.35 (−148.28–96.13)
5 HR-HPV types^b^	565	28	4.96	558	10	1.79	63.83 (23.30–84.33)
7 HR-HPV types^c^	565	44	7.79	558	39	6.99	10.25 (−41.35–43.21)
8 HR-HPV types^d^	565	45	7.96	558	41	7.35	7.74 (−44.08–41.06)
13 HR-HPV types^e^	565	72	12.74	558	67	12.01	5.77 (−33.28–33.46)
Any type	565	98	17.35	558	89	15.95	8.04 (−23.79–31.77)

a Confidence intervals (CI) were calculated using the two-step approach, which did not account for dependence.

b 5 HR-HPV types: 16, 18, 31, 33, 45.

c 7 HR-HPV types: 16, 18, 31, 33, 45, 52, 58.

d 8 HR-HPV types: 16, 18, 31, 33, 35, 45, 52, 58.

e 13 HR-HPV types: 16, 18, 31, 33, 35, 39, 45, 51, 52, 56, 58, 59, 68. *Abbreviations*: mITT, modified Intention-to-treat; HPV, human papilloma virus; HR-HPV, high risk human papilloma virus.

### 3.2 The protective efficacy in the baseline HPV-positive population

In the cohort positive for any baseline HPV type, the control group exhibited one instance of HPV-16 infection, one instance of HPV-18 infection, and two instances of HPV-16/18 infection after 9 years; the vaccine group had one case of HPV-16 infection, no HPV-18 infections, and one case of HPV-16/18 infection. The analysis of the protective efficacy of the HPV vaccine against HPV-16, 18, and 16/18 in the baseline HPV-positive population after 9 years was 25.00% (95% CI: −5 787.26 to 99.04), 100.00% (95% CI: −2 825.00 to 100.00), and 62.49% (95% CI: −620.48 to 99.36), respectively.

The evaluation of the protective efficacy of the HPV vaccine against infection related to non-vaccine types was conducted on individuals who were initially positive for one vaccine type-DNA but negative for the other vaccine type-DNA at baseline (positive for HPV-16 or HPV-18, excluding co-infections). Within the baseline HPV-16-infected cohort, one instance of HPV-18 infection emerged in the control group after 9 years, whereas no HPV-16/18 infections were detected in the vaccinated group. Conversely, in the baseline HPV-18-infected cohort, no occurrences of HPV-16 infection were identified in the control group after 9 years, while one case of HPV-16 infection was found in the vaccine group.

The influence of highly homologous HPV types on the vaccine's protective efficacy against HPV-16/18-related infections at baseline was further explored in individuals infected with vaccine-type nucleotides. The findings indicated that among female individuals who tested positive for any non-16 type Alpha-9 series HPV at baseline, no HPV-16 and 18-related infections were found after 9 years of vaccination; in the female population positive for any non-18 type Alpha-7 series HPV type at baseline, no HPV-16 or HPV-18-related infections were found in either the vaccine or control group after 9 years (Table 3).

**Table 3:** Efficacy of HPV-related infection protection in baseline HPV-positive women.

HPV type	Control group			Vaccine group			Vaccine efficacy (95% CI, %)
	Total number	Number of cases	Rate (%)	Total number	Number of cases	Rate (%)	
HPV-positive population at baseline							
HPV-16	63	1	1.59	84	1	1.19	25.00 (−5 787.26–99.04)
HPV-18	63	1	1.59	84	0	−	100.00 (−2 825.00–100.00)
HPV-16/18	63	2	3.17	84	1	1.19	62.50 (−620.48–99.36)
HPV-16 positive population at baseline							
HPV-16	10	0	−	17	0	−	−
HPV-18	10	1	10.00	17	0	−	100.00 (−2 194.12–100.00)
HPV-16/18	10	1	10.00	17	0	−	100.00 (−2 194.12–100.00)
HPV-18 positive population at baseline							
HPV-16	2	0	−	3	1	33.33	−
HPV-18	2	0	−	3	0	−	−
HPV-16/18	2	0	−	3	1	33.33	−
HPV-16 negative and any non-16 type Alpha-9 species DNA positive population^a^							
HPV-16	23	0	−	24	0	−	−
HPV-18	23	0	−	24	0	−	−
HPV-16/18	23	0	−	24	0	−	−
HPV-18 negative and any non-18 type Alpha-7 species DNA positive population^a^							
HPV-16	2	0	−	9	0	−	−
HPV-18	2	0	−	9	0	−	−
HPV-16/18	2	0	−	9	0	−	−

a HPV Alpha-7 species refer to HPV-18, 39, 45, 59, 68, and Alpha-9 species refer to HPV-16, 31, 33, 35, 52, 58.

## 4 Discussion

The results of mid-term and final analyses of a bivalent HPV vaccine based on an *E. coli* expression system were disseminated in 2020 and 2022.^8^^,^^9^ The mid-term analysis indicated that the protective efficacy of the per-protocol set (PPS) dataset against HPV-16, HPV-18, and HPV-16/18 infections was 69.8% (95% CI: 57.4–79.0), 64.2% (95% CI: 45.5–77.1), and 79.0% (95% CI: 62.3–89.1), respectively. Subsequent follow-up studies demonstrated that the protective efficacy of the bivalent HPV vaccine against HPV-16, HPV-18, and HPV-16/18 infections remained stable, showing no decline when compared with the mid-term analysis data.

Analysis of the ITT and mITT datasets indicated that the bivalent HPV vaccine can effectively protect against HPV-16/18 infections (ITT: VE = 87.34, 95% CI: 46.17–98.59; mITT: VE = 87.37, 95% CI: 46.26–98.59). In comparison to the mid-term and final analyses, VE slightly improved. Several factors may account for this observation: First, the LTFU study selected one of the sites registered in phase III clinical trials, and the distribution of HPV DNA subtypes is different in different regions. Additionally, compared with the sample size of the mid-term analysis, the sample size of the LTFU was relatively small. The mITT dataset used in the mid-term and final analysis also incorporated antibody data, whereas the LTFU study relied solely on HPV DNA results. In addition, this study exclusively used the baseline data and the 2022 follow-up results from the cohort, omitting cases from the intermediate follow-up period. Furthermore, those in the mid-term and final analyses of this cohort comprised individuals who had not achieved virus clearance within a short timeframe. Nine years later, as a result of vaccination efforts and ongoing follow-up screening, a substantial number of previous cases had been resolved. Consequently, the infection status of the vaccinated group at this juncture more accurately reflects new infections.

This study, constrained by a limited sample size, suggests that the bivalent HPV vaccine based on *E. coli* may exhibit some cross-protective effects against HPV-31, 33, and 45; however, these effects were not significant. By contrast, the HPV-16/18 AS04-adjuvanted vaccine demonstrates a more robust cross-protective response, attributable to its adjuvant properties^11^; the cross-protection of the other vaccines is relatively weaker.

This study evaluated the protective efficacy of the HPV vaccine among individuals who tested positive for HPV DNA. At baseline, 147 participants (13.09%) were identified as having an infection from any HPV type. By the ninth year of follow-up, the overall HPV infection rate within the entire cohort had increased to 16.65%. Among those with a positive baseline HPV DNA result, the protective efficacy of the vaccine against HPV-16, 18, and HPV-16/18 was 25.00% (95% CI: −5 787.26 to 99.04), 100.00% (95% CI: −2 825.00 to 100.00), and 62.50% (95% CI: −620.48 to 99.36), respectively. Furthermore, nucleotide homology exists within the L1 region among HPV Alpha-9 (HPV-31, 33, 35, 52, 58) and Alpha-7 subtypes (HPV-39, 45, 59, 68), as well as between the L1 regions of HPV-16 and HPV-18. The L1 homology between HPV-31 and HPV-16 is 83%, while that between HPV-45 and HPV-18 is 88%.^12^ In this study, there were no instances of infections with non-vaccine types from the Alpha-7 or Alpha-9 series observed at baseline, including HPV-16/18.

This study represents the inaugural LTFU investigation of the bivalent HPV vaccine (produced in *E. coli*), assessing its protective efficacy against HPV infection 9 years after vaccination. This study had three limitations. First, this study did not analyze endpoints other than HPV infection. Second, the sample size presents certain constraints that may affect the generalizability of the findings. Third, the statistical analysis was confined to cases documented during the LTFU phase, which may limit the comprehensiveness of the conclusions drawn. In conclusion, the bivalent HPV vaccine (*E. coli*) demonstrated long-term protective efficacy against HPV-16 and HPV-18 infections for at least 9 years.

## Funding

This study was supported by the 10.13039/501100003345CAMS Innovation Fund for Medical Sciences (Grant Number: CAMS 2021-I2M-1-004). It also received support from the Tencent Sustainable Social Value Inclusive Health Lab and the ChongQing Tencent Sustainable Development Foundation through the “Comprehensive Prevention and Control Demonstration Project for Eliminating Cervical Cancer and Breast Cancer in Low Health Resource Areas of China” (Project Number: SD20240904145730).

## CRediT authorship contribution statement

**Xinhua Jia:** Writing – original draft, Investigation. **Shangying Hu:** Writing – review & editing. **Xuefeng Kuang:** Investigation. **Youlin Qiao:** Writing – review & editing.
